# A prospective study of twinning and perinatal mortality in urban Guinea-Bissau

**DOI:** 10.1186/1471-2393-12-140

**Published:** 2012-12-05

**Authors:** Morten Bjerregaard-Andersen, Najaaraq Lund, Frida Staarup Jepsen, Luis Camala, Margarida Alfredo Gomes, Kaare Christensen, Lene Christiansen, Dorte Møller Jensen, Peter Aaby, Henning Beck-Nielsen, Christine Stabell Benn, Morten Sodemann

**Affiliations:** 1 Bandim Health Project, INDEPTH Network, Apartado 861, 1004, Bissau Codex, Guinea-Bissau; 2 Department of Infectious Diseases, Odense University Hospital, Sdr. Boulevard 29, 5000, Odense C, Denmark; 3 Research Center for Vitamins and Vaccines (CVIVA), Statens Serum Institute, Artillerivej 5, 2300, Copenhagen S, Denmark; 4 Department of Maternity, National Hospital Simão Mendes, Bissau, Guinea-Bissau; 5 The Danish Twin Registry, Epidemiology, Institute of Public Health, University of Southern Denmark, J.B. Winsløwsvej 9, 5000, Odense C, Denmark; 6 Department of Clinical Biochemistry and Pharmacology, Odense University Hospital, Sdr. Boulevard 29, 5000, Odense C, Denmark; 7 Department of Clinical Genetics, Odense University Hospital, Sdr. Boulevard 29, 5000, Odense C, Denmark; 8 Department of Endocrinology, Odense University Hospital, Kløvervænget 6, 5000, Odense C, Denmark

## Abstract

**Background:**

Despite twinning being common in Africa, few prospective twin studies have been conducted. We studied twinning rate, perinatal mortality and the clinical characteristics of newborn twins in urban Guinea-Bissau.

**Methods:**

The study was conducted at the Bandim Health Project (BHP), a health and demographic surveillance site in Bissau, the capital of Guinea-Bissau. The cohort included all newborn twins delivered at the National Hospital Simão Mendes and in the BHP study area during the period September 2009 to August 2011 as well as singleton controls from the BHP study area. Data regarding obstetric history and pregnancy were collected at the hospital. Live children were examined clinically. For a subset of twin pairs zygosity was established by using genetic markers.

**Results:**

Out of the 5262 births from mothers included in the BHP study area, 94 were twin births, i.e. a community twinning rate of 18/1000. The monozygotic rate was 3.4/1000. Perinatal mortality among twins vs. singletons was 218/1000 vs. 80/1000 (RR = 2.71, 95% CI: 1.93-3.80). Among the 13783 hospital births 388 were twin births (28/1000). The hospital perinatal twin mortality was 237/1000.

Birth weight < 2000g (RR = 4.24, CI: 2.39-7.51) and caesarean section (RR = 1.78, CI: 1.06-2.99) were significant risk factors for perinatal twin mortality. Male sex (RR = 1.38, CI: 0.97-1.96), unawareness of twin pregnancy (RR = 1.64, CI: 0.97-2.78) and high blood pressure during pregnancy (RR = 1.77, CI: 0.88-3.57) were borderline non-significant. Sixty-five percent (245/375) of the mothers who delivered at the hospital were unaware of their twin pregnancy.

**Conclusions:**

Twins had a very high perinatal mortality, three-fold higher than singletons. A birth weight < 2000g was the strongest risk factor for perinatal death, and unrecognized twin pregnancy was common. Urgent interventions are needed to lower perinatal twin mortality in Guinea-Bissau.

## Background

Newborn twins constitute a high risk group [1,2], as they are much more likely to suffer from low birth weight, prematurity and distress during labor and delivery [2-4]. While this is known for all settings, including high-income countries [2,5], the effect becomes more pronounced in areas with inadequate pre- and postnatal care [1].

In Sub-Saharan Africa twins account for a significant part of perinatal and infant deaths [1,6]. According to the WHO the average perinatal mortality is 56/1000 [7], although mortality rates of 155-248/1000 are reported for twins [2,8,9], i.e. a several-fold increase. At the same time twinning is more common in Sub-Saharan Africa, with a regional estimate of 20/1000 [1]. Since monozygotic (MZ) twinning is considered constant at around 3-4/1000, this increase is mainly due to frequent dizygotic (DZ) twinning [1].

Twins therefore remain an important target group for interventions in Sub-Saharan Africa and require particular attention. In this regard it is especially important to identify risk factors for twin mortality. However, until now relatively few twin studies have been carried out in Sub-Saharan Africa [1,2,4,10]. Of the studies done [2,6,8-16], most have examined hospital records retrospectively [1,2]. Therefore there is a need of *prospective* cohort studies focusing specifically on the epidemiological and clinical aspects of twinning.

This is the first study on a twin cohort established in Guinea-Bissau. The aim was to determine the MZ and DZ twinning rates and perinatal mortality, both at community and hospital levels. Furthermore, we wanted to compare newborn twins and singletons clinically and identify risk factors for perinatal twin death.

## Methods

### Bandim health project

The study was conducted at the Bandim Health Project (BHP) in Bissau, the capital of Guinea-Bissau. Guinea-Bissau is a small, low-income country in West Africa. The BHP is a health and demographic surveillance site and a member of the INDEPTH (International Network for the Demographic Evaluation of Populations and Their Health in Developing Countries).

### Bandim health project study area

The BHP monitors a study area of approximately 100,000 individuals in Bissau, the capital of Guinea-Bissau. All individuals are registered with an ID-number, age, sex, ethnic group, socio-economic characteristics and twin status. All pregnancies and births are registered. The information is updated through routine censuses. Once a newborn is identified, the child is followed with regular home visits.

In this paper the BHP study area is denoted “study area”. The term “community” is used in the results and discussion parts to describe events among study area children. We only included children identified by the BHP before birth, as early deaths might not be reported retrospectively, and this could lead to an underestimation of the true mortality.

### Community control cohort

All study area singletons served as a comparison group for twins from the study area when assessing perinatal mortality.

### National Hospital Simão Mendes

The BHP registers all deliveries at the maternity ward at the National Hospital Simão Mendes, situated in the centre of Bissau, - two kilometers from the study area. The hospital serves the whole capital and the surrounding areas. A substantial part of the mothers from the study area give birth here. The mothers pay for giving birth at the hospital.

At delivery the BHP registers sex, birth weight, the presentation of the child, the mode of delivery and vital status. Information regarding the mother’s age, ethnic group, previous pregnancies and socio-economic status is also collected, and all live children are monitored by the BHP until discharge. Events occurring at the hospital maternity ward are denoted “hospital”.

### Twin inclusions at the hospital

All newborn twins, both from the study area and outside, were included at the hospital during 24 months between September 2009 and August 2011. Every day, including weekends and holidays, a BHP assistant registered new twin deliveries and filled out a specific twin questionnaire in addition to the general birth registration. The questionnaire included the mother’s name, age, ethnicity, residence, twins in the family and awareness of twin pregnancy. Antenatal pregnancy cards were examined when available. These standardized cards are filled out at antenatal consultations. The collected information included gestational age, illness during pregnancy, blood pressure (BP), edemas, and whether the pregnancy was considered “high risk”. BP at arrival was obtained from the hospital’s clinical birth records. Maternal height was measured with standard measuring tape, weight was registered using a Seca scale for adults which was calibrated regularly. Middle upper arm circumference (MUAC) was measured with non-stretchable measuring tape.

Newborn twins were weighed on an electronic Seca scale for children. A clinical examination was done by a neonatal nurse. Gestational maturity was assessed with the Ballard score, which includes both neurological and physical maturation signs. It is considered valid and reproducible, also for very premature children [17].

After discharge twins from outside the study area were accompanied home to facilitate follow-up visits. Twins from the interior of the country (i.e. outside the capital Bissau) were not monitored. Twins from the study area were automatically monitored by the BHP registration system.

### Follow-up

The first follow-up visit was undertaken at two months of age. Data collection included vital status, morbidity, anthropometry and vaccinations.

### Clinical control cohort

Unmatched singleton controls were included at the hospital between January 2010 and August 2011. The controls included singleton deliveries among study area mothers. Initially, the inclusion frequency was every 7th study area birth, but from February 2011 this was adjusted to every 5th study area birth to increase the number of controls. The singletons served as controls when studying clinical differences between twins and singletons at the hospital.

### Overlap between study area and hospital births

Forty-six percent of all mothers from the study area gave birth at the hospital, i.e. an overlap exists between the study area (community) and the hospital data. Among all hospital births study area children accounted for 17%, the rest were either from other parts of Bissau or the interior of the country.

### HIV testing

HIV testing was offered to women delivering at the hospital. A rapid test Determine (Abbot Diagnostics, Maidenhead, United Kingdom) was used. Positive results were confirmed with SD Bioline HIV-1/2 (Standard Diagnostics, Kyonggi-do, South Korea). Antiretroviral treatment was provided to prevent vertical transmission.

### Zygosity sub-study

A zygosity sub-study was carried out during the first 20 months. Once maternal consent was obtained, heel blood was collected on filter paper from live same-sex twin pairs. The samples were stored frozen in Guinea-Bissau and afterwards transported frozen to Odense University Hospital, Denmark, for genetic analyses. Zygosity was established using 12 highly polymorphic microsatellite markers. Concordance in all markers indicates a greater than 99.8% probability of monozygosity [18].

### Ethics

Informed consent either by signature or fingerprint was obtained from the mother prior to interview, examination and analyses. The study was approved by the National Health Ethics Committee in Guinea-Bissau. The Central Ethical Committee in Denmark gave consultative approval.

### Definitions

The term “perinatal deaths” denotes both stillbirths and early neonatal deaths [19]. This paper only reports on the perinatal period, i.e. until the first seven days after birth. Low birth weight (LBW) was defined as birth weight below 2500g, very low birth weight (VLBW) as birth weight below 2000g [2].

Prematurity was defined as birth before 37 weeks (260 days) of gestation [17]. Birth before 22 weeks of gestation was considered an abortion [19]. At the hospital no distinction was made between miscarriages and stillbirths due to the frequent lack of information about gestational age. Nor was any distinction made between “fresh” and “macerated” stillbirths.

The number of pregnancies included the current pregnancy. Births where one twin was born at home and the other at the hospital were considered hospital births.

### Statistical methods

Data was entered using dBase 5.0 software (dataBased Inc, Vestal, NY, USA). Statistical analyses were done using STATA software (Stata Corporation, College Station, TX, USA). Comparisons of categorical variables were done using Poisson regression with robust variance estimates and expressed as probabilities (P) or relative risks (RR) [20]. Absolute differences (Diff) in continuous variables were assessed using linear regression. Risk factors for perinatal mortality were analysed by Poisson regression, by using robust variance estimates and expressed as RR. All analyses were adjusted for the interdependency (clustering) of outcomes within twin pairs. Ratios were calculated with 95% confidence intervals.

The risk factors for perinatal twin death were selected after a literature review [2,5,8,10,15,21-28]. Infant risk factors were male sex, low birth weight, prematurity, long intertwin delivery interval (>30 minutes), breech presentation, caesarean section and being the second twin. Maternal risk factors were age < 18 years, primigravida, HIV infection, high BP (>140/90) during pregnancy and unawareness of twin pregnancy. Lack of education and being unmarried were included as socio-economic indicators. The rainy season was included as an exogenous risk factor. The selected risk factors were included in a multivariate analysis, including the univariately non-significant risk factors. Prematurity was not included in the multivariate model due to frequently missing or unreliable gestational age. A long intertwin delivery interval was considered a risk factor for the second twin only and therefore not part of the multivariate model either.

We report the tripling rate, but have otherwise excluded triplets as they represent a special group and constitute a very small fraction of multiple gestations [10,29].

## Results

### Twinning rate and perinatal mortality in the study area

During the study period the BHP identified 6875 newborn children in the study area. Out of those children 1409 (60 twins and 1349 singletons) were not registered until after birth and therefore excluded. The perinatal mortality in the excluded group was 50/1000 (3/60) for twins and 8/1000 (11/1349) for singletons.

**Figure 1:**
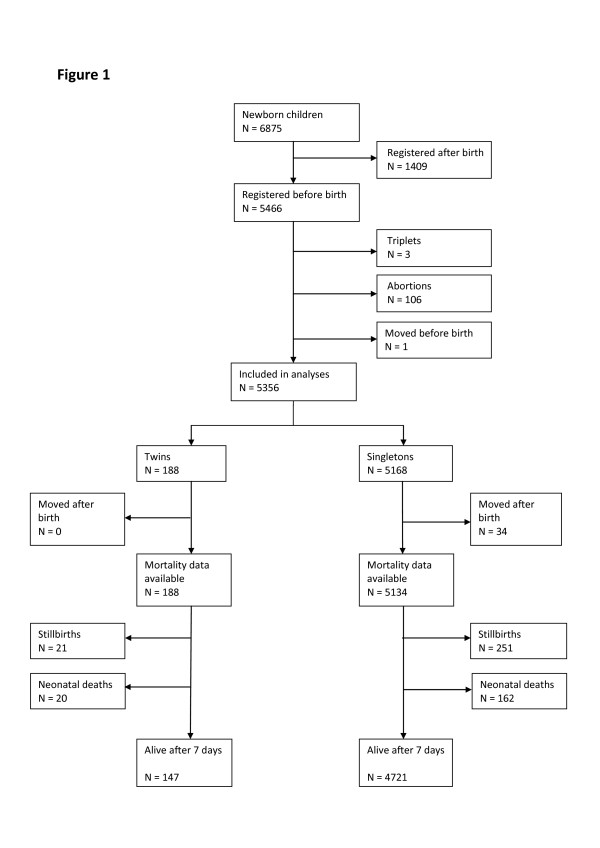
**Study area children born between September 2009 and August 2011.** The figure includes children born both at the hospital and outside the hospital.

Of 5466 children that were registered before birth, three triplets (one set) were excluded. Furthermore, 106 abortions and one case of movement before birth were also excluded due to difficulty in assessing twin status of the pregnancy. Thus, 5356 children (5262 births) were included in the analyses, i.e. 188 twins (94 twin births) and 5168 singletons. The community twinning rate was 18/1000 while the tripling rate was 0.2/1000 (Figure 1).

Among study area twins there were 21 stillbirths and 20 early neonatal deaths, i.e. 41 perinatal deaths. Among singletons there were 251 stillbirths and 162 early neonatal deaths, i.e. 413 perinatal deaths. Thirty-four movements after birth among singletons were censored from the mortality analysis. Thus, the community perinatal mortality in twins and singletons was 218/1000 and 80/1000 (RR = 2.71, CI: 1.93-3.80), respectively, see Figure 1 and Table 1. No maternal deaths among the 94 twin mothers were observed.

**Table 1:** Mortality among study area children born between September 2009 and August 2011

	**Twins**^**1**^	**Singletons**^**1**^	**RR (CI)**
**Perinatal death**	41/188 (218/1000)	413/5134 (80/1000)	RR = 2.71 (1.93-3.80)
Perinatal death by sex*			
Male	24/102 (235/1000)	217/2624 (83/1000)	RR = 2.85 (1.91-4.23)
Female	17/86 (198/1000)	196/2509 (78/1000)	RR = 2.53 (1.54-4.14)
Perinatal death by birth weight**			
Birth weight ≥ 2500g	4/49 (82/1000)	126/2934 (43/1000)	RR = 1.90 (0.77-4.71)
Birth weight < 2500g	18/93 (194/1000)	74/342 (216/1000)	RR = 0.89 (0.51-1.56)

^1^Cases of movement after birth have been censored.

*Sex was missing for one child.

**Birth weight was missing for 1904 children.

The table includes children born both at the hospital and outside the hospital.

### Twinning rate and perinatal mortality at the hospital

**Figure 2:**
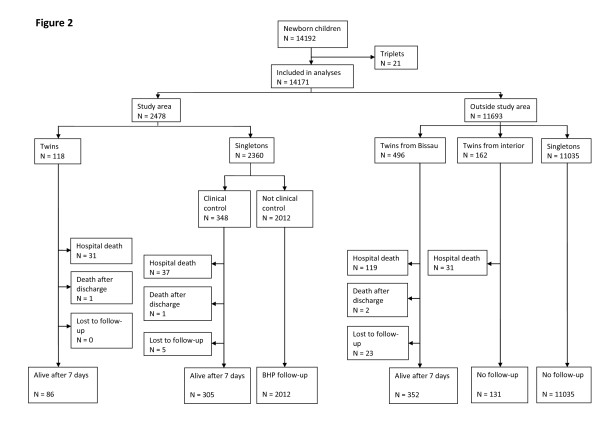
**Hospital births between September 2009 and August 2011.** The figure includes children from both inside and outside the study area.

At the hospital 14192 children were registered. After excluding 21 triplets (seven sets) 14171 children (13783 births) were included in the analyses, i.e. 776 twins (388 twin births) and 13395 singletons. The hospital twinning rate was 28/1000 while the tripling rate was 0.5/1000 (Figure 2).

Out of the 776 twins 118 were from the study area, 496 from the rest of Bissau and 162 from the interior (Figure 2).

Among the 776 twins born there were 184 perinatal deaths, including 181 deaths at the hospital and 3 deaths after discharge. The 181 hospital deaths consisted of 110 stillbirths and 71 early neonatal deaths. The 184 perinatal deaths were almost evenly distributed between first and second born twins, i.e. 89 (48%) and 95 (52%), respectively. The perinatal mortality for hospital born twins was 237/1000 (Figure 2). There were five maternal deaths among the 388 twin mothers (13/1000).

### Study area children born at the hospital

Forty-six percent (2478/5356) of the study area children were born at the hospital. For twins the proportion was higher than for singletons, i.e. 62.8% (118/188) vs. 45.7% (2360/5168).

### Zygosity sub-study

**Figure 3:**
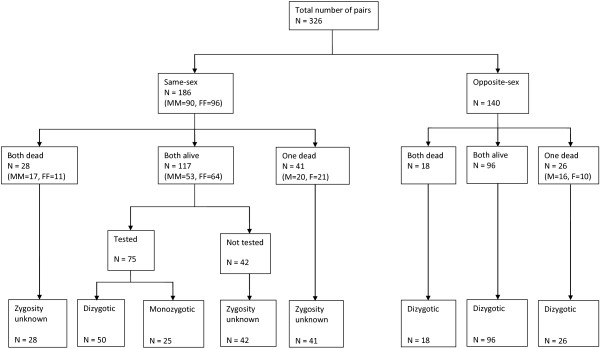
**Zygosity sub-study.** The flowchart includes 326 twin pairs (652 children) born at the hospital. Sex has been included in parenthesis (M = Male, F = Female).

A zygosity sub-study was conducted for 326 twin pairs (652 children) born at the hospital (Figure 3). The study included 186 same-sex pairs and 140 opposite-sex pairs. The same-sex pairs consisted of 117 live pairs, 41 pairs with one twin dead, and 28 pairs with both twins dead.

Zygosity was established genetically in 75/117 (64%) of the live same-sex pairs, while 42/117 (36%) did not have blood collected as the mother refused to give consent, had left or the twins were VLBW. In the latter case blood collection was attempted during follow-up. Blood was also not collected from pairs with one or both twins dead. Among the 75 live same-sex pairs with genetically established zygosity, 25 were MZ and 50 DZ. Thus, out of the tested same-sex pairs 33% (25/75) were MZ while 67% (50/75) were DZ. Assuming the zygosity distribution was the same among all same-sex twins, we would have approximately 61 MZ same-sex pairs (33% of 186). Thus, out of the total number of 326 twin pairs, MZ twins would account for 19% (61/326). Assuming a true community twinning rate of 18/1000, the MZ rate would therefore be 3.4/1000 (0.19*18/1000).

### Clinical characteristics of twins vs. singletons at the hospital

Among the 2360 study area singletons born at the hospital 348 clinical controls were selected (Figure 2). Twins were on average 791g (CI: 706–877) lighter than singletons, and VLBW was far more common among twins (RR = 7.51, CI: 4.42-12.8) (Table 2). Twins also had lower gestational age (Diff = 12 days, CI: 4–20) and were more often premature (RR = 1.7, CI: 1.2-2.5). The Ballard score was significantly lower among twins (Diff = 4.6, CI: 3.9-5.4). Twins were more often born by caesarean section (RR = 2.41, CI: 1.66-3.49) and were older at discharge (Diff = 2.7 days, CI: 2.2-3.2).

**Table 2:** Clinical comparison of 776 twins and 348 singletons born at the hospital

	**Twins**	**Singletons**	**RR (CI), P or Diff (CI)**
	**N = 776**	**N = 348**	
**Infant**			
Male sex	373/776 (48.1%)	175/348 (50.3%)	RR = 0.96 (0.84-1.09)
Birth weight; g (SD)	2271 (632)	3063 (583)	Diff = 791 (706–877)
Birth weight category			
> = 2500g	305/763 (40.0%)	302/343 (88.0%)	RR = 0.45 (0.41-0.51)
2000-2499g	224/763 (29.4%)	27/343 (7.9%)	RR = 3.73 (2.54-5.47)
<2000g	234/763 (30.7%)	14/343 (4.1%)	RR = 7.51 (4.42-12.8)
Gestational age at birth; days (SD)	261 (35)	273 (28)	Diff = 12 (4–20)
Prematurity	81/198 (41%)	38/162 (23%)	RR = 1.7 (1.2-2.5)
Ballard score (SD)	37.9 (5.6)	42.6 (3.2)	Diff = 4.6 (3.9-5.4)
Intertwin delivery interval; minutes (SD)	14.0 (31.0)	-	-
Long intertwin delivery interval (>30 minutes)	54/686 (7.9%)	-	-
Breech presentation at birth	123/773 (16%)	2/347 (0.6%)	RR = 27.6 (6.9-111)
Caesarean section	177/773 (22.9%)	33/347 (9.5%)	RR = 2.41 (1.66-3.49)
Age at discharge; days (SD)	4.2 (5.1)	1.5 (2.1)	Diff = 2.7 (2.2-3.2)
**Mother**			
Age; years (SD)	26.5 (5.5)	24.8 (5.8)	Diff = 1.7 (0.9-2.6)
Age < 18 years	12/382 (3.1%)	33/347 (9.5%)	RR = 0.33 (0.17-0.63)
Ethnicity			
Balante	104/387 (26.9%)	29/347 (8.4%)	P < 0.001
Fula	82/387 (21.2%)	80/347 (23.1%)	
Pepel	58/387 (15.0%)	95/347 (27.4%)	
Mandinka	45/387 (11.6%)	14/347 (4.0%)	
Other	98/387 (25.3%)	129/347 (37.1%)	
No. of pregnancies (SD)	3.26 (1.76)	2.44 (1.58)	Diff = 0.82 (0.57-1.06)
Primigravida	65/382 (17.0%)	127/346 (36.7%)	RR = 0.46 (0.36-0.60)
Previous perinatal death	44/319 (13.8%)	42/219 (19.2%)	RR = 0.72 (0.49-1.06)
BMI after birth; kg/m^2^ (SD)	23.1 (4.18)	23.7 (4.41)	Diff = 0.59 (÷0.05-1.24)
MUAC; mm (SD)	250 (30.9)	257 (35.8)	Diff = 7.1 (2.1-12.1)
Family history of twinning	160/377 (42.4%)	98/345 (28.4%)	RR = 1.49 (1.21-1.83)
Mother is twin herself	15/377 (4.0%)	15/345 (4.3%)	RR = 0.92 (0.45-1.84)
Father is twin himself	36/377 (4.8%)	13/345 (3.8%)	RR = 1.27 (0.63-2.55)
Mother has other twins	28/377 (7.5%)	3/345 (0.87%)	RR = 8.54 (2.62-27.9)
Mother of mother has twins	96/377 (25.5%)	64/345 (18.6%)	RR = 1.37 (1.04-1.82)
Mother of father has twins	60/377 (15.9%)	45/345 (13.0%)	RR = 1.22 (0.85-1.74)
HIV infection	22/311 (7.1%)	12/282 (4.3%)	RR = 1.66 (0.84-3.30)
Lack of schooling	167/380 (43.9%)	67/345 (19.4%)	RR = 2.26 (1.77-2.89)
Unmarried	207/382 (54.2%)	172/346 (49.7%)	RR = 1.09 (0.95-1.25)
**Pregnancy**			
High BP (>140/90) at hospital arrival	30/149 (20.1%)	8/120 (6.7%)	RR = 3.02 (1.44-6.35)
High BP (>140/90) during pregnancy	14/322 (4.3%)	4/315 (1.3%)	RR = 3.42 (1.14-10.3)
Edemas	27/318 (8.5%)	1/316 (0.3%)	RR = 26.8 (3.7-197)
Illness during pregnancy	53/375 (14.1%)	26/346 (7.5%)	RR = 1.88 (1.20-2.94)
High risk pregnancy	17/264 (6.4%)	8/280 (2.9%)	RR = 2.25 (0.99-5.14)
Unawareness of twin pregnancy	245/375 (65.3%)	-	-

Twin mothers were older than singleton mothers (Diff = 1.7 year, CI: 0.9-2.6) and had a higher number of pregnancies (Diff = 0.82, CI: 0.57-1.06). Maternal MUAC was significantly lower among twin mothers (Diff = 7.1 mm, CI: 2.1-12.1), and post-partum BMI tended to be lower (Diff = 0.59 kg/m^2^, CI: ÷0.05-1.24).

Twinning was more frequent among the ethnic group Balantas (P < 0.001), and also if the mother (RR = 8.54, CI: 2.62-27.9) or mother of the mother (RR = 1.37, CI: 1.04-1.82) had a previous history of birthing twins. Hypertension (RR = 3.42, CI: 1.14-10.3) was more prevalent among twin mothers during pregnancy. No significant difference was observed in the prevalence of maternal HIV infection (RR = 1.66, CI: 0.84-3.30). Sixty-five percent (245/375) of twin mothers reported unawareness of twin pregnancy. At antenatal consultations only 6.4% (17/264) were classified as high risk pregnancies. For singleton mothers the frequency of high risk pregnancy was 2.9% (8/280) (RR = 2.25, CI: 0.99-5.14). Of the 17 twin mothers classified as high risk pregnancies, 7 (41%) were still unaware they were expecting twins.

### Caesarean sections at the hospital

In total, 94 caesarean sections were performed. In 83 of the cases both twins were delivered this way, while for 11 births only the second twin was delivered by caesarean section. Thus, a total of 177 twins were delivered by caesarean section. The use of internal podalic version for twin deliveries was not recorded, but the method is occasionally applied.

### Twins from inside vs. outside study area at the hospital

Study area twin mothers were older compared to twin mothers from outside the study area (P = 0.004). Twins from the study area had lower birth weight (P = 0.03) and had also lower Ballard score (P = 0.03). Otherwise no significant differences were observed.

### Risk factors for perinatal twin death at the hospital

Table 3 examines risk factors for perinatal twin death at the hospital. In the multivariate analysis birth weight < 2000g (RR = 4.24, CI: 2.39-7.51) and caesarean section (RR = 1.78, CI: 1.06-2.99) were significant risk factors. Male sex (RR = 1.38, CI: 0.97-1.96), unawareness of twin pregnancy (RR = 1.64, CI: 0.97-2.78) and high BP during pregnancy (RR = 1.77, CI: 0.88-3.57) were borderline non-significant.

**Table 3:** Risk factors for perinatal death among 776 twins born at the hospital

	**N**^**1**^	**Univariate analysis**	**Multivariate analysisN**^**2**^** = 464**
		***RR (CI)***	***Adjusted RR (CI)***
**Infant risk factors**			
Male sex	776	1.30 (1.00-1.71)	1.38 (0.97-1.96)
Birth weight category	763		
> = 2500g (ref)		1.00	1.00
2000-2499g		1.15 (0.69-1.92)	1.10 (0.57-2.09)
<2000g		4.48 (3.00-6.70)	4.24 (2.39-7.51)
Prematurity*	198	3.78 (2.05-6.97)	-
Long intertwin delivery interval**	343	1.47 (0.79-2.73)	-
Breech presentation at birth	773	0.97 (0.66-1.42)	1.26 (0.72-2.12)
Caesarean section	773	0.99 (0.68-1.44)	1.78 (1.06-2.99)
Second twin	776	1.07 (0.88-1.29)	0.97 (0.69-1.36)
**Maternal risk factors**			
Age < 18 years	764	1.26 (0.59-2.68)	1.53 (0.54-4.31)
Primigravida	764	1.52 (1.07-2.15)	0.80 (0.44-1.46)
HIV infection	622	1.06 (0.57-2.00)	1.09 (0.66-1.80)
High BP (>140/90) during pregnancy	644	1.97 (1.03-3.79)	1.77 (0.88-3.57)
Unawareness of twin pregnancy	750	1.89 (1.26-2.85)	1.64 (0.97-2.78)
**Socio-economic risk factors**			
Lack of schooling	760	0.97 (0.71-1.33)	0.89 (0.58-1.34)
Unmarried	764	0.90 (0.66-1.23)	1.13 (0.73-1.74)
**Exogenous risk factors**			
Rainy season	776	0.85 (0.63-1.16)	0.91 (0.59-1.40)

^1^ Number of observations for each variable in the univariate model.

^2^ Number of children included in the multivariate model.

* Prematurity was only included in the univariate model due to a high number of missing data.

** Interval between delivery of the first and the second twin of more than 30 minutes. Only analyzed for the second twin and therefore not included in multivariate model.

Prematurity was not included in the multivariate model due to the frequently missing or unreliable gestational age. However, the univariate analysis showed a strong correlation (RR = 3.78, CI: 2.05-6.97).

## Discussion

### Main observations

The twinning rate was moderately high, i.e. 18/1000 at community level and 28/1000 at the hospital. The MZ rate was 3.4/1000 in the community. Perinatal twin mortality was very high with community and hospital rates of 218/1000 and 237/1000, respectively. In the community the RR of perinatal death among twins vs. singletons was 2.71 (CI: 1.93-3.80).

Newborn twins had on average 791g lower birth weight than singletons. VLBW was the strongest risk factor for perinatal twin death. Maternal unawareness of twin pregnancy was common, and only a fraction of the twin mothers had been registered as high risk pregnancies antenatally.

### Strengths and weaknesses

Considering that newborn twins constitute a substantial and very vulnerable group in Sub-Saharan Africa, a surprisingly small number of twin studies are available. A literature search revealed only few other *prospective* mortality datasets [2,10]. Most twin studies are based on retrospective analyses of hospital records and hence do not report community data. To our knowledge, this is the first cohort study to specifically target twin mortality; it has a large sample size and presents both community and hospital data. It is also one of the first to genetically determine the zygosity distribution.

The study has a number of limitations. Follow-up was only carried out within Bissau and is therefore missing for twins from the interior of the country. This could underestimate the hospital perinatal mortality. However, only very few deaths were registered immediately after discharge.

The zygosity sub-study was only done on live pairs. Hence, we have no zygosity status of same-sex pairs with one or both twins dead. MZ twins, who are often monochorionic, are at higher risk of death *in utero*[3,30]*.* We may therefore have underestimated the MZ rate. Furthermore, for VLBW twins blood collection was postponed until follow-up. As LBW twins are more often MZ [3,31] and some twins were either dead or not located upon follow-up, this could mean that more MZ pairs were missed.

We did not monitor antenatal consultations in detail. Apart from the pregnancy cards, we have limited data on the nurses’ and midwives’ abilities to identify and monitor twin pregnancies.

At the hospital, twin mothers came from both inside and outside the study area, while the singleton controls were selected from the study area only. This could bias comparisons of clinical characteristics (Table 2). Though smaller differences were observed, most variables showed no significant differences. Hence, the bias is probably small.

Gestational age was calculated by the last menstrual date. This method is considered somewhat imprecise [5], and the prematurity estimates should therefore be interpreted cautiously. Consequently, maturity was also assessed using the Ballard score. As gestational age was often unavailable, we did not include prematurity in the multivariate analysis. This is an important limitation. However, due to a presumed overlap between LBW and prematurity [4] we believe that the multivariate analysis still provides important information.

We excluded children who were only registered after birth in the study area. This was done to avoid the bias of retrospective registration of stillbirths and early neonatal deaths, which is often unreliable and may result in the underestimation of the true mortality. The perinatal mortality was much lower in the excluded group.

Unfortunately, no distinction was made between “fresh” and “macerated” stillbirths. This limits our ability to distinguish between intra-partum and ante-partum deaths. It is, however, likely that many stillbirths were fresh and therefore labor related [32].

Birth weight was often missing in the community data. The main reason was birth at home. This could bias the comparison of twin vs. singleton mortality by birth weight (Table 1), as it would predominantly include children born in health institutions [11].

Though the cost of delivery in itself is quite small (~3 USD), costs can rapidly accumulate if medicine, utensils or in particular a cesarean section (~100 USD) are needed. Hence, financial constraints in emergency situations are likely to cause a mortality increase.

### Consistency with previous findings

The community twinning rate of 18/1000 was similar to neighboring Gambia, Burkina Faso and Senegal [10,15,33]. The hospital rate of 28/1000 probably reflects an overestimation due to selective referral of twin mothers [1], a fact which is also illustrated in the community data by more twin (63%) than singleton (46%) mothers giving birth at the hospital. It should be emphasized that the estimates represent the “natural twinning rate” as *in vitro* fertilization is very uncommon in Guinea-Bissau.

Twinning refers to two separate phenomena [1]. While MZ twinning is the result of one fertilized egg dividing into two identical embryos, DZ twins are caused by the fertilization of two ova in the same cycle. The MZ rate is fairly constant around the world and independent of maternal age and parity. From Sub-Saharan Africa this has however mainly been theoretically confirmed by Weinberg’s differential rule, where the frequency of DZ twins is twice the number of opposite-sexed pairs [34]. Thus, in our study, the proportion of DZ twins at the hospital would be (2*140/326) * 100% = 86% and the MZ proportion would only be 14%, which is somewhat different from the genetic estimate of 19% MZ twins. A 19% MZ proportion would confirm a MZ rate of 3-4/1000 (0.19*18/1000) [1]. Studies from the Gambia and Burkina Faso have estimated MZ proportions of around 30% [10,15].

Unlike the MZ rate, DZ twinning varies globally, with the highest rate observed in Sub-Saharan Africa. It is dependent on age, parity and ethnicity [1]. Genetic disposition also seems important since twinning was eightfold more likely among previous twin mothers in our study. This raises the question why DZ twinning is so common in Africa? Given the high mortality, fewer children may actually result from twin pregnancies. This should, at least in theory, cause a gradual genetic selection against twinning. Since this is not the case, there may be important health benefits associated with DZ twinning [1], as recently confirmed in the Gambia [35].

This study found perinatal twin mortality to be very high. Other African studies from Burkina Faso, the Gambia, Congo and Malawi have found overall perinatal mortality rates of 64-79/1000 [23], while WHO’s regional estimate is 56/1000 [7]. In Europe and North America, much lower average rates are observed, i.e. 7-8/1000 [7]. Hence, a community perinatal mortality of 218/1000 for twins in our setting is disturbing and demands attention. Twin studies from Nigeria have reported perinatal mortality rates between 155-186/1000 [8,9], while in rural Gambia an early neonatal twin mortality of 114/1000 is described [10]. A small study from rural Malawi found a perinatal twin mortality of 248/1000 [2].

Likewise, it is worrying that the twin mortality rates at the hospital (237/1000) and in the community (218/1000) were almost similar, as one would expect a lower mortality given hospital delivery. A possible explanation is that in Guinea-Bissau twin pregnancies often remain unrecognized until delivery. Furthermore, many mothers give birth at home or in health centers with limited obstetrical expertise. This may cause delays in the referral of complicated twin deliveries, causing many twin mothers to arrive too late at the hospital. Transportation issues (especially at night), lack of money and delays due to availability of hospital staff may aggravate this [32].

It should be noted that while the RR of 2.71 for twin vs. singleton death is actually lower than in some high-income countries, e.g. 4.22 in the US [5], the high “background” singleton mortality (80/1000) makes newborn twins very vulnerable in Guinea-Bissau. Thus, in absolute terms (deaths per thousand twin and singleton births), the difference is much higher.

Clinically, twins were smaller and more premature than singletons [2,10,15] and discharged later. Twin mothers were older and had higher parity [1,3]. Ethnicity was also important [1], as twinning was more prevalent among the Balantas. Twin mothers were more ill during pregnancy, including suffering from hypertensive disorders [2].

The strongest predictor for perinatal twin death was VLBW [2,10], which was associated with a fourfold increase in mortality. As twins account for 20-31% of all LBW newborns in West Africa [26,36], this has considerable impact on the overall perinatal mortality. Prematurity is another well described risk factor among twins [2,10]. The univariate analysis revealed a more than threefold increase in perinatal mortality.

Maternal unawareness of twin pregnancy tended to be a risk factor. This is worrying given the large number of women (65% of twin mothers), and it suggests gaps in proper health examinations during antenatal care. Even if a twin pregnancy was in fact noted, the mother may not have been properly counseled or referred to hospital birth. The fact that 20% of twin mothers arrived with hypertension could also indicate problems in diagnosing and treating common pregnancy disorders. Hypertension during pregnancy tended to confer a higher risk of perinatal twin death, presumably due to pre-eclampsia [25].

Caesarean section was associated with nearly twofold higher twin mortality. This may be due to the fact that the caesarean sections were mainly emergency procedures in case of serious fetal or maternal distress [37]. However, fatal delays are also likely to play a role. Delays may exist in getting to the hospital, finding the means to pay for a caesarean section and in hospital staff realizing the problem and taking appropriate action [32].

The tendency for higher mortality among male twins could reflect increased early neonatal mortality among males in general [24]. Surprisingly, maternal HIV infection was not a risk factor though this has been reported by others [38].

Finally, a previous study by our group found that twins are more likely to suffer from hypothermia during the first day of life [39]. It is therefore possible that newborn twins do not always receive adequate monitoring of adverse conditions such as hypothermia, septicemia and signs of prematurity in our setting.

### Implications

The very high perinatal twin mortality in Guinea-Bissau calls for immediate action. At hospital level, specialized training in handling twin deliveries has proved effective [2], both intra-partum and post-partum. Though hospital resources are scarce, training in simple neonatal resuscitative procedures can lower death rates markedly [40].

Although antenatal care was not the focus, our data suggest that better identification and monitoring of twin pregnancies are needed. Sixty-five percent of the mothers reported to be unaware of their twin pregnancy. Of those where pregnancy cards were available, only 6% of the twin mothers had been classified as high risk pregnancies. Limiting premature twin births should be a key priority [26], and previous studies show that clinical examination alone can detect the majority of twin pregnancies [41,42].

There are several reasons why twins should receive particular attention, apart from being a high risk group. First, provided the antenatal and hospital services gradually improve, the relative proportion of twin deaths in overall perinatal mortality is likely to increase [1], as mortality in twins is more difficult to prevent [43]. Secondly, due to fetal distress and other adverse perinatal outcomes, newborn twins are at high risk of long term sequelae (e.g. neurological) even if they survive [4]. As systematic follow-up is difficult in low-income countries, improved management of twin pregnancies is essential. Thirdly, twins can be used as a proxy of antenatal care, e.g. the percentage of twin pregnancies recognized prior to delivery. This is important in settings where simple monitoring tools are needed.

## Conclusions

Perinatal twin death remains an unnoticed problem in Sub-Saharan Africa, despite a high frequency of twin births. In Guinea-Bissau, the perinatal twin mortality rate was very high (218/1000 in the community), and there is an acute need for interventions to reduce this, both in the primary sector and at hospital level. Focus should be on improving hospital conditions for twin deliveries as well as on better identification and monitoring of twin pregnancies at the health centers.

## Competing interests

We declare that none of the authors have any competing interests.

## Authors’ contributions

MB-A, MS, CSB and PAA designed the study. MB-A, NL, FSJ, LC and MAG supervised inclusions and clinical routines in Guinea-Bissau. LChr conducted the genetic analyses in Denmark. KC, DMJ, HB-N co-designed the study and offered advice throughout the process. All authors approved of the final manuscript.

## Pre-publication history

The pre-publication history for this paper can be accessed here:

http://www.biomedcentral.com/1471-2393/12/140/prepub
